# Inequalities in smoking among pregnant women in North West London

**DOI:** 10.1093/pubmed/fdad040

**Published:** 2023-04-23

**Authors:** Ana-Catarina Pinho-Gomes, Edward Mullins

**Affiliations:** The George Institute for Global Health, Imperial College London, Fourth Floor Translation and Innovation Hub, London W12 0BZ, UK; Institute of Health Informatics, University College London, London NW1 2DA, UK; The George Institute for Global Health, Imperial College London, Fourth Floor Translation and Innovation Hub, London W12 0BZ, UK; Department of Metabolism, Digestion and Reproduction, Imperial College London, London W12 0BZ, UK

**Keywords:** smoking, pregnancy, inequalities

## Abstract

**Background:**

London has the lowest smoking prevalence among pregnant women in England. However, it was unclear whether the low overall prevalence masked inequalities. This study investigated the prevalence of smoking among pregnant women in North West London stratified by ethnicity and deprivation.

**Methods:**

Data regarding smoking status, ethnicity and deprivation were extracted from electronic health records collected by maternity services at Imperial Healthcare NHS Trust between January 2020 and August 2022.

**Results:**

A total of 25 231 women were included in this study. At the time of booking of antenatal care (mean of 12 weeks), 4% of women were current smokers, 17% were ex-smokers and 78% never smokers. There were marked differences in the smoking prevalence between ethnic groups. Women of Mixed—White and Black Caribbean ethnicity and White Irish women had the highest prevalence of smoking (12 and 9%, respectively). There was an over 4-fold increase in the prevalence of smoking between the most and the least deprived groups (5.6 versus 1.3%).

**Conclusions:**

Even in a population with an overall low prevalence of smoking in pregnancy, women experiencing deprivation and from certain ethnic backgrounds have a high smoking prevalence and hence are the most likely to benefit from smoking cessation interventions.

## Introduction

Smoking in pregnancy is associated with an increased risk of adverse outcomes, such as stillbirth, preterm birth and low birth weight,^1^^,^^2^ and smoking cessation can reduce those risks to a level almost comparable to non-smokers.^3^ In England, 9.7% of women were smoking at the time of delivery in 2021.^4^ Although this represents a decrease from about 14% in 2011, it is markedly short of the target set by the Department of Health of 6% or lower to be achieved by 2022.^5^ Considering current trends, it may take a further 10 years from 2021 to achieve that target. In addition, among those who were smokers at the time they attended their first midwife appointment, only 36% stopped smoking in pregnancy.^4^ This illustrates the societal and personal difficulties surrounding smoking cessation and emphasizes the importance of supporting pregnant women in quitting smoking, as they are likely to be more receptive and motivated during pregnancy due to the perceived benefits for them and their babies.^6^

Although London has the lowest smoking prevalence among pregnant women in England (about 4–5%),^4^ we hypothesized that the low overall prevalence could mask important inequalities according to ethnicity and deprivation with implications for smoking cessation service delivery. Therefore, this study aimed to investigate the prevalence of smoking among pregnant women in North West London stratified by ethnicity and deprivation.

## Methods

Data were obtained from electronic health records collected by maternity services between January 2020 and August 2022 at Imperial College Healthcare NHS Trust, which serves the population of North West London. Data were extracted for demographic variables and smoking at the first time of contact with maternity services. Smoking status was self-reported and validated with measurement of exhaled carbon monoxide. We calculated the prevalence of smoking stratified by deprivation and ethnicity. Deprivation was categorized into fifths of the Index of Multiple Deprivation (IMD) for the postcode of residence. Ethnicity was self-reported according to the pre-defined categories available on the electronic health records system. Both variables were collected at the time of first contact with maternity services. This study was approved by the Yorkshire & The Humber—South Yorkshire Research Ethics Committee, reference 19/YH/0435.

## Results

**Fig. 1:**
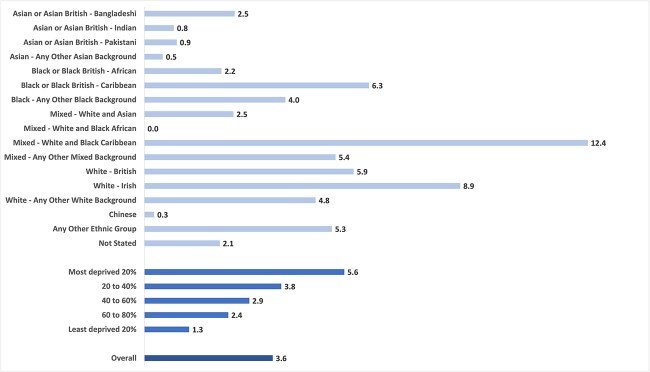
Smoking prevalence (%) in early pregnancy in maternity services at Imperial Healthcare NHS Trust between January 2020 and August 2022 (*N* = 25 231 women).

A total of 25 231 women were included in this study, with a mean age of 32 years (Table 1). The largest ethnic group was any other White background (26%), followed by White British (16%) and any other Asian background (13%). Pregnant women were distributed across the entire range of the IMD fifths, with 21% in the most deprived fifth and 34% the second least deprived fifth. At the time of booking of antenatal care (mean of 12 weeks), 4% of the women were current smokers, 17% were ex-smokers and 78% never smokers. There were marked differences in the smoking prevalence between ethnic groups (Fig. 1). For instance, the prevalence of smoking at booking for antenatal care was 12% for women of White and Black Caribbean ethnicity and 9% for White Irish women. There was also a stark deprivation gradient with an over 4-fold increase in the prevalence of smoking between the most and the least deprived IMD fifths.

**Table 1:** Characteristics of the study population

**Age (years), mean (SD)**	32.0 (5.5)
**Ethnicity, *N* (%)**	
Asian or Asian British—Bangladeshi	237 (0.9)
Asian or Asian British—Indian	1694 (6.7)
Asian or Asian British—Pakistani	660 (2.6)
Asian—any other Asian Background	3301 (13.1)
Black or Black British—African	1571 (6.2)
Black or Black British—Caribbean	857 (3.4)
Black—any other Black background	1642 (6.5)
Mixed—White and Asian	200 (0.8)
Mixed—White and Black African	145 (0.6)
Mixed—White and Black Caribbean	233 (0.9)
Mixed—any other mixed background	615 (2.4)
White—British	4170 (16.5)
White—Irish	282 (1.1)
White—any other White background	6617 (26.2)
Chinese	366 (1.5)
Any other ethnic group	38 (0.2)
Not stated	2603 (10.3)
**Deprivation, *N* (%)**	
Least deprived 20%	1591 (6.3)
Deprivation 20–40%	8586 (34.0)
Deprivation 40–60%	6045 (24.0)
Deprivation 60–80%	3822 (15.1)
Most deprived 20%	5187 (20.6)
**Smoking status, *N* (%)**	
Current smoker	909 (3.6)
Ex-smoker	4351 (17.2)
Never smoked	19 751 (78.3)
Unknown	220 (0.9)
**Gestation age at booking (weeks), mean (SD)**	12.1 (5.7)

## Discussion

### Main findings

This study demonstrated that in a population of pregnant women living in North West London, with a broad distribution of deprivation and ethnic diversity, there are important differences in the prevalence of smoking at the time of booking antenatal care. These illustrate how an overall low local prevalence of smoking (4%) can hide inequalities between socioeconomic and ethnic groups, with a prevalence of smoking 3- to 4-fold above the average and similar to the highest national values in certain groups.

### What is already known on this topic

The importance of smoking cessation during pregnancy has been highlighted by recent guidelines on tobacco smoking published by the National Institute for Health and Care Excellence (NICE) in England, which recommend using financial incentives (i.e. vouchers) to encourage smoking cessation during pregnancy in addition to nicotine replacement therapy and behavioural support.^7^^,^^8^ These guidelines also recommended e-cigarettes to support smoking cessation for adults but not pregnant women as evidence is lacking on their efficacy and safety in pregnancy.^9^ Since these guidelines were published, a large UK-based trial demonstrated that financial vouchers (i.e. LoveToShop shopping vouchers redeemable in many retail outlets) reduced by almost 3-fold the odds of smoking in pregnancy, even if most women relapsed after giving birth.^10^ Pregnant women experiencing deprivation have an increased risk of adverse pregnancy and birth outcomes due to a constellation of risk factors.^11^ Unfortunately, they are also the most likely to continue smoking in pregnancy.^4^ Therefore, they have the most to benefit from smoking cessation and they are likely to be more receptive to financial incentives.^12^ Despite compelling evidence on their cost-effectiveness and NICE recommendation, implementation of financial vouchers as incentives to smoking cessation in pregnancy in England remains patchy. For instance, although in Greater Manchester they have been routinely offered since 2018, in Greater London they are not yet available. Implementation of NICE recommendations consistently across the country may help addressing inequalities in smoking in pregnancy.

### What this study adds

Our findings of stark inequalities illustrate the importance of disaggregating data by ethnicity and socioeconomic group. The markedly higher prevalence of smoking among women living in the most deprived areas in comparison with those living in the most affluent areas is concerning because women experiencing deprivation are more likely to have other risk factors for adverse pregnancy outcomes, such as obesity, cardiometabolic diseases and poor mental health.^13–15^ Smoking further elevates the risk of adverse birth outcomes, such as preterm birth and intrauterine growth restriction, which have adverse and lifelong consequences for the offspring.^16^ Children growing in deprived neighbourhoods are also exposed to multiple risk factors for poor health, such as lack of green space, poor living conditions and food poverty.^17^ Therefore, reducing the avoidable detrimental consequences of smoking for mothers and their offspring, particularly those experiencing deprivation, is crucial.

### Limitations of this study

This study has some limitations. First, it relied on routinely collected electronic health records rather than on data collected purposefully for research. This meant that a small fraction of data was missing (e.g. 10% for ethnicity and 1% for smoking). Second, our study population is specific to North West London and findings may not be generalizable elsewhere. Third, there is a substantial overlap between ethnic minorities and deprivation. However, we found very different prevalence of smoking between the ethnic groups experiencing similar levels of deprivation. Fourth, we did not have data on smoking status at the time of birth to investigate inequalities in smoking cessation during pregnancy.

## Conclusion

There are marked inequalities in smoking based on ethnic background and deprivation among pregnant women in North West London. Financial incentives, as recommended by NICE guidelines, may improve smoking cessation during pregnancy and reduce inequalities even in areas with low overall smoking prevalence in pregnancy, preventing the detrimental and lifelong consequences of smoking for the offspring.

## Data Availability

All data are available upon request from the corresponding author.

## Notes

**Ana-Catarina Pinho-Gomes**, NIHR Clinical Lecturer in Public Health Medicine

**Edward Mullins**, Clinical Senior Lecturer and Honorary Consultant Obstetrician
